# Composition of "Ruspini's Styptic."

**Published:** 1831-03

**Authors:** A. T. Thomson


THE LONDON
### Medical and Physical Journal.

No. 385, VOL. LXVI.] MARCH 1831. [No. 57, *New Series.*

For many fortunate discoveries in medicine, and for the detection of numerous errors, the world is indebted to the rapid circulation of Monthly Journals; and there never existed any work to which the Faculty, in Europe and America, were under deeper obligations than to the *Medical and Physical Journal of London*, now forming a long but invaluable series.—RUSH.

### ORIGINAL PAPERS, AND CASES,

### OBTAINED FROM PUBLIC INSTITUTIONS AND OTHER AUTHENTIC SOURCES.

### RUSPINI'S STYPTIC.

*Composition of "Ruspini's Styptic."* By Dr. A. T. THOMSON.

*To the Editor of the London Medical and Physical Journal.*

DEAR SIR: Among the multitude of pretended remedies which are sold, in this country, under the name of patent medicines, a few have been employed by regular physicians, surgeons, and general practitioners, from a conviction that they prove beneficial in the cases for which they have been prescribed; demonstrating the liberality of the properly educated practitioner over the ignorant, narrow-minded, yet generally too highly patronised, quack. One of these medicines is Ruspini's styptic, which has been found very useful in stemming those hemorrhagies that cannot be commanded by the needle and ligatures: such, for instance, as occur from the intestinal canal, the bladder of urine, and other internal cavities. But, useful as this medicine is in such cases, the dose required to be given is so large as to render it the most expensive remedy, not excepting the alkaloids and their salts, which has been introduced for many years. I felt that I should be doing a service to the profession, therefore, by analyzing this styptic, and thus enabling practitioners to employ its active principle at a cheap rate. The following is the result of this inquiry.

1. A part of a bottle of Ruspini's styptic was evaporated at a temperature under 120° of Fahrenheit: the residue was a crystallized salt, in whitish, acicular crystals, which impressed a weak, sour taste on the palate, and dissolved entirely in distilled water; the solution slightly reddening litmus paper.

2. Into a portion of the aqueous solution (1), largely diluted, a solution of Persulphate of Iron was dropped; a beautiful dark blue colour was instantly developed.

3. Another portion of the solution (1,) being tested with Ammonia in solution, a deep orange-red colour was evolved, which, after twenty-four hours, became paler, and verging to green.

4. A third portion, treated in the same manner with Liquor Potassæ, afforded a permanent reddish orange colour, which deepened by exposure to the air.

5. Lime water, added to the solution (1), afforded a greenish precipitate, which was afterwards dissolved by an excess of the test.

From the effects of these tests, there is no doubt that Gallic acid is the active principle of this styptic; minute quantities of Opium and Sulphate of Zinc, which I have ascertained it, also, contains, being of no account, from the smallness of their proportions; and, as the vehicle is alcohol, with a small quantity of rose water, to give it odour, the price which has been charged for the quantity contained in the bottles sold for 8s. 6d. each, cannot be regarded in any other light than that of a barefaced imposition on the public. Alcohol dissolves one fourth of its weight of Gallic acid: an excellent styptic, therefore, may be at any time extemporaneously prescribed. In cases of hemorrhagies from the bladder, the addition of some Gallic acid to a tincture of Uva Ursi, will be found to answer every indication which can be expected from the employment of astringents in this disease. The Gallic acid is taken into the circulation, and passes undecomposed through the kidnies; so that it is thus applied to the bleeding vessels of the bladder, or those of the prostate gland, or of the intestines, in the same manner as when a dossil of lint, soaked in a solution of it, is applied to bleeding vessels on the surface of the body.

I remain, dear sir, yours faithfully,

A. T. THOMSON.

*3, Hinde street, Manchester square;*
*20th January,* 1831.

